# Ferroptosis in Alzheimer’s disease: molecular mechanisms and advances in therapeutic strategies

**DOI:** 10.3389/fnins.2025.1673315

**Published:** 2026-01-12

**Authors:** Ze Zhou, Yiting Zhang, Siyi Liu, Haixia Tang, Lianhao Yang, Yanming Lu, Jiaobao Liao, Shuowei Zhang, Zukun Chen, Ling Yang

**Affiliations:** Yunnan University of Chinese Medicine, Kunming, Yunnan, China

**Keywords:** Alzheimer’s disease, ferroptosis, iron homeostasis, glutathione peroxidase 4, lipid peroxidation, immune regulation, precision medicine

## Abstract

Alzheimer’s disease (AD) is a progressive neurodegenerative disorder characterized primarily by the continuous decline of cognitive functions. Its pathogenesis involves complex, multidimensional interactions among various molecular pathways. In recent years, ferroptosis, a regulated form of iron-dependent cell death, has emerged as a crucial contributor to AD progression. Ferroptosis is defined by the accumulation of lipid peroxides and inactivation of glutathione peroxidase 4 (GPX4), and is typically initiated in the context of disrupted iron homeostasis, aberrant lipid metabolism, and mitochondrial dysfunction in the brain. This review comprehensively delineates the molecular mechanisms underlying dysregulated iron metabolism in AD and proposes an integrative “iron–lipid–energy–inflammation” axis as a pathological framework. Particular attention is given to the GPX4 signaling pathway as a central hub linking lipid peroxidation, mitochondrial damage, and immune responses. Moreover, ferroptosis can propagate through intercellular mechanisms involving the release of damage-associated molecular patterns (DAMPs), dysregulation of immune checkpoints, and exosome-mediated signaling, collectively driving microglial activation, T-cell infiltration, and blood–brain barrier disruption, culminating in systemic immune imbalance. We further evaluate multiple therapeutic strategies targeting ferroptosis, including iron chelators, antioxidants, GPX4 activators, and lipoxygenase inhibitors. Based on emerging evidence, we propose a precision medicine approach that incorporates ferroptosis subtyping, multi-omics analysis, and targeted delivery systems. Ferroptosis represents a promising frontier for early diagnosis and intervention in AD, potentially enabling the development of causality-oriented, mechanism-based therapies.

## 1 Introduction

Alzheimer’s disease (AD) is the most prevalent neurodegenerative disorder among the elderly, characterized by progressive impairments in cognition, behavior, and daily functioning, with short-term memory loss often presenting as an early symptom (Pei et al., 2025). The hallmark pathological features of AD include the accumulation of β-amyloid (Aβ) peptides forming senile plaques and hyperphosphorylated tau aggregating into neurofibrillary tangles (NFTs), accompanied by synaptic dysfunction, neuronal loss, and chronic neuroinflammation (Agostinho et al., 2010; Winblad et al., 2016). In recent years, accumulating evidence suggests that oxidative stress, mitochondrial dysfunction, and disturbances in iron homeostasis serve as upstream triggers driving these pathological cascades (Luo et al., 2019; Pal et al., 2022). Iron is an essential trace element in the central nervous system (CNS), playing critical roles in oxygen transport, mitochondrial ATP production, and neurotransmitter biosynthesis (Belaidi and Bush, 2016). Notably, pathological iron accumulation has been consistently observed in the hippocampus and cortical regions of AD patients. Excessive iron promotes hydroxyl radical (•OH) generation via the Fenton reaction, thereby exacerbating lipid peroxidation and oxidative injury, which in turn facilitates Aβ aggregation, tau hyperphosphorylation, and neuronal degeneration (Pei et al., 2025; Ward et al., 2014). Disruption of iron homeostasis can initiate ferroptosis, a regulated form of cell death characterized by iron-dependent lipid peroxidation. This process is driven by the inactivation of glutathione peroxidase 4 (GPX4) and the pathological accumulation of lipid hydroperoxides (LPOs) within cellular membranes (Stockwell et al., 2017; Xie et al., 2023; Li X. et al., 2024). Preclinical studies in Alzheimer’s disease models have demonstrated that pharmacological inhibition of ferroptosis with small-molecule inhibitors such as ferrostatin-1 or liproxstatin-1 alleviates lipid peroxidation, neuronal death, and cognitive decline (Bao et al., 2021; Majerníková et al., 2024). With over 50 million individuals currently affected by dementia worldwide—a number projected to double by 2050—there is a critical need for novel, mechanism-based interventions, particularly in the absence of curative therapies (Sexton et al., 2022). Ferroptosis represents a promising mechanistic axis for therapeutic innovation in AD. Future research should prioritize the identification of actionable targets within the ferroptotic pathway, assess the regulatory potential of natural compounds and traditional medicines on iron metabolism, and accelerate translational efforts to incorporate ferroptosis-modulating strategies into clinical practice (Table 1).

**Table 1:** Comparison between ferroptosis and other forms of programmed cell death.

Feature category	Ferroptosis	Apoptosis	Pyroptosis	Necroptosis	Autophagic cell death
Inducing factors	Iron overload, lipid peroxidation, GSH depletion, GPX4 inactivation (Jiang et al., 2021)	DNA damage, p53 activation, intrinsic/extrinsic stress (Taylor et al., 2008)	Pathogen infection, TLR/NLR activation, inflammasome (Kayagaki et al., 2015)	TNF-α, virus infection, Caspase-8 deficiency (Luo et al., 2022)	Nutrient deprivation, hypoxia, ROS accumulation, anticancer drugs (Mizushima and Komatsu, 2011)
Key molecules/pathways	GPX4, ACSL4, SLC7A11, ALOX15, FTH1 (Dixon et al., 2012)	Caspase-3, Bcl-2 family, Cyt c, p53 (Green et al., 2009)	Caspase-1, GSDMD, NLRP3, IL-1β (Liu et al., 2024)	RIPK1, RIPK3, MLKL (Luo et al., 2022)	Beclin1, LC3-II, ATG5, mTOR (Kourtis and Tavernarakis, 2009)
Morphological features	Mitochondrial shrinkage, cristae loss, increased membrane density, no nuclear fragmentation (Yang et al., 2014)	Nuclear condensation, DNA fragmentation, membrane integrity retained (Green et al., 2009)	Cell swelling, membrane pore formation, foam-like structure (Liu et al., 2024)	Cell swelling, membrane rupture, cytoplasmic release (Chen et al., 2024)	Autophagosome accumulation, organelle degradation, vacuolation (Gao et al., 2019)
Iron dependency	Yes (iron-dependent lipid peroxidation) (Tang et al., 2021)	No	No	No	Partial (iron-regulated autophagy pathway) (Wang et al., 2014)
Immunogenicity/inflammation	High, releases DAMPs and activates microglia/immune cells (Fan et al., 2021)	Low, immunologically silent (Valentini et al., 2023)	Highly pro-inflammatory, IL-1β release (Amand et al., 2023)	Moderate inflammation (Luo et al., 2022)	Context-dependent, partial immune modulation (Gao et al., 2019)
Inhibitors	Liproxstatin-1, Ferrostatin-1, Deferoxamine (Zilka et al., 2017; Chu et al., 2023)	Z-VAD-FMK, Bcl-2 mimetics (Bruce et al., 2025; Tatton and Olanow, 1999)	VX-765, MCC950, Caspase-1 inhibitors (Jiang et al., 2020; Huang et al., 2021)	Necrostatin-1, GSK872 (Liu M. et al., 2022; Zhang et al., 2017)	3-MA, Bafilomycin A1, Chloroquine (Yang et al., 2013; Wang Y. et al., 2022)
Related diseases	AD, Parkinson’s, cancer, cardiomyopathy (Yan et al., 2021; Zhou et al., 2024; Yang et al., 2022; Elmore, 2007)	Neurodegeneration, cancer, development (Mustafa et al., 2024; Krasovec et al., 2022; Shi et al., 2017)	Autoinflammatory and infectious diseases (Magnani et al., 2022; Vandenabeele et al., 2010)	Neurodegeneration, ischemia, viral infection (Mitroshina et al., 2022; Daniels et al., 2017; Zaarour et al., 2021)	Neural injury, cancer, metabolic diseases (Khandia et al., 2019; Reiss et al., 2018)

This table outlines the major distinctions between ferroptosis and other forms of regulated cell death, including apoptosis, necroptosis, and pyroptosis. Ferroptosis is defined by iron-dependent lipid peroxidation, impaired GPX4 activity, and an expansion of the labile iron pool. In contrast, apoptosis is characterized by caspase activation and DNA fragmentation, whereas necroptosis and pyroptosis are initiated through receptor-driven or inflammatory signaling pathways. Recent studies also indicate that excess intracellular iron can modulate necroptosis and pyroptosis, suggesting that disrupted iron homeostasis is not exclusive to ferroptosis. Even so, ferroptosis remains the most strongly iron-dependent mode of cell death and exhibits a uniquely amplified lipid-oxidation cascade. Given that Alzheimer’s disease is marked by abnormal iron accumulation, reduced antioxidant defenses, and heightened vulnerability to ferroptotic injury, the distinctions summarized in this table are important for understanding how multiple cell death pathways may intersect during AD progression.

## 2 Brain iron metabolism

### 2.1 Brain iron metabolism and its regulatory mechanisms

Iron is an essential trace element required for maintaining normal function of the central nervous system (CNS). It participates in critical metabolic processes including myelin synthesis, neurotransmitter production, and mitochondrial electron transport (Silva and Faustino, 2015). The homeostasis of cerebral iron is tightly regulated through a complex network of transport and storage proteins, involving transferrin (Tf), non-transferrin-bound iron (NTBI), transferrin receptor (TfR1), divalent metal transporter 1 (DMT1), ferroportin 1 (FPN1), ferroxidases such as hephaestin (HEPH) and ceruloplasmin (CP), and intracellular storage protein ferritin (Baringer et al., 2023). Iron enters the brain primarily through two distinct pathways across the blood–brain barrier (BBB). The predominant mechanism involves Tf/TfR1-mediated endocytosis, where transferrin-bound Fe^3+^ binds to TfR1 on endothelial cells, is internalized into endosomes, acidified, and subsequently released into the cytosol via DMT1-mediated transport. In contrast, NTBI can cross the BBB via low-efficiency, non-specific diffusion processes (Yu and Chang, 2019). Iron efflux from the brain is primarily mediated by FPN1, the only known cellular iron exporter. FPN1 functions in concert with ferroxidases such as HEPH and CP, which oxidize Fe^2+^ to Fe^3+^, enabling its safe release into the systemic circulation (Zhou and Tan, 2017). Intracellular iron homeostasis is governed by the iron regulatory protein–iron-responsive element (IRP–IRE) system. This post-transcriptional regulatory mechanism modulates mRNA stability and translation of key iron-handling genes. Under low-iron conditions, IRPs bind to IREs in the untranslated regions (UTRs) of target mRNAs, stabilizing TfR1 and DMT1 transcripts while suppressing translation of ferritin and FPN1. In contrast, under high-iron conditions, IRPs undergo conformational inactivation and dissociate from IREs, allowing increased synthesis of ferritin and FPN1 and reduced iron uptake (Sanchez et al., 2007; Muckenthaler et al., 2017). Specifically, IRPs binding to IREs at the 5′-UTR suppress translation of ferritin, FPN1, and amyloid precursor protein (APP), while IRP binding at the 3′-UTR stabilizes mRNAs of TfR1 and DMT1, thereby promoting iron uptake under conditions of deficiency (Mai et al., 2023). Hepcidin, a liver-derived peptide hormone, also exerts a crucial negative feedback role in cerebral iron regulation. Elevated hepcidin levels suppress the expression of FPN1, TfR1, and DMT1, thereby limiting iron entry into the brain. Hepcidin expression itself is influenced by systemic iron levels, inflammatory cytokines, and hypoxic conditions (Su et al., 2022). Systemically, iron is absorbed primarily in the duodenum, where it enters intestinal epithelial cells via DMT1. DMT1 expression is upregulated during iron deficiency and downregulated under iron overload conditions. Intercellular transport of iron predominantly relies on the Tf/TfR1 system, while ferritin serves as the principal intracellular reservoir and buffer against labile iron fluctuations (Jain et al., 2023; Galluzzi et al., 2018; Goto et al., 2021). Under physiological conditions, iron undergoes reversible Fe^2+^/Fe^3+^ cycling that enables redox-dependent enzymatic reactions essential for CNS function. This delicate balance sustains oxidative metabolism while preventing uncontrolled radical generation. When iron homeostasis is perturbed, excess Fe^2+^ catalyzes Fenton chemistry to produce highly reactive hydroxyl radicals (•OH), triggering lipid peroxidation and cellular injury. These redox-driven events create a biochemical milieu that predisposes neurons to ferroptosis (Dixon et al., 2012; Guo et al., 2013).

### 2.2 Ferroptosis and its regulatory pathways

**Figure 1:**
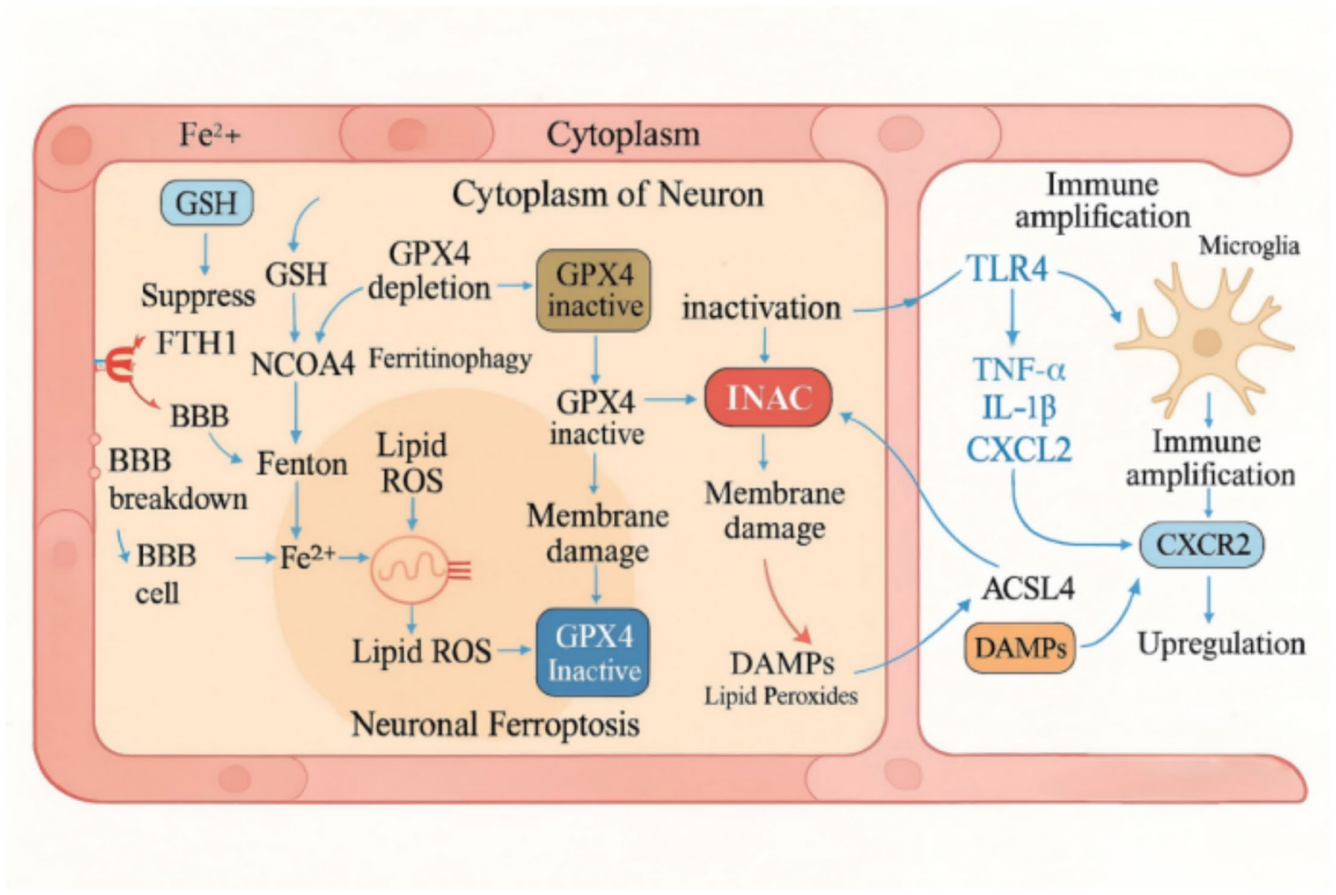
Mechanistic illustration of ferroptosis-mediated neuronal damage in AD. This schematic illustrates the core molecular events linking brain iron dysregulation to ferroptotic neurodegeneration in AD. Excess Fe^2+^ derived from transferrin (Tf)–transferrin receptor 1 (TfR1) uptake or ferritinophagy expands the labile iron pool (LIP), catalyzing Fenton reactions that generate hydroxyl radicals (•OH). The resulting lipid reactive oxygen species (ROS↑) initiate peroxidation of polyunsaturated fatty acids (PUFAs) via ACSL4- and LPCAT3-mediated remodeling of phospholipids. Under physiological conditions, the glutathione peroxidase 4 (GPX4)–glutathione (GSH) axis and the cystine/glutamate antiporter System Xc^−^ (SLC7A11/xCT) detoxify lipid peroxides and maintain redox balance. In AD, inhibition or depletion of GPX4 and SLC7A11—together with increased ACSL4, ferritin degradation, and impaired NRF2-antioxidant signaling—exacerbate lipid peroxidation and neuronal ferroptosis. Non-canonical regulators such as mitochondrial dysfunction, ER stress, and inflammatory cytokines (TNF-α, IFN-γ) further amplify oxidative injury. Pharmacological inhibitors of ferroptosis (ferrostatin-1, liproxstatin-1, deferoxamine [DFO]) and metal-protein-attenuating compounds (PBT2) are shown as protective interventions targeting iron overload and lipid peroxidation. ACSL4, acyl-CoA synthetase long-chain family 4; AD, Alzheimer’s disease; DFO, deferoxamine; GPX4, glutathione peroxidase 4; GSH, glutathione; IFN-γ, interferon-gamma; LIP, labile iron pool; LPCAT3, lysophosphatidylcholine acyltransferase 3; NRF2, nuclear factor erythroid 2–related factor 2; PUFA, polyunsaturated fatty acid; ROS, reactive oxygen species; SLC7A11/xCT, cystine/glutamate transporter; Tf, transferrin; TfR1, transferrin receptor 1.

Ferroptosis, a distinct form of regulated cell death first defined by Dixon et al. (2012), is characterized by iron-dependent accumulation of lipid peroxides, distinguishing it from classical cell death modalities such as apoptosis, necrosis, and autophagy, ferroptosis exhibits a stronger dependence on intracellular iron than other regulated cell death pathways; however, emerging evidence indicates that iron dysregulation can also modulate pyroptosis and necroptosis (Tang et al., 2021; Zilka et al., 2017). Morphologically, ferroptotic cells exhibit shrunken mitochondria, increased membrane density, loss of cristae, and rupture of the outer mitochondrial membrane. Biochemically, it is marked by disrupted iron metabolism, accumulation of lipid hydroperoxides (LPO), and depletion of intracellular glutathione (GSH). Notably, ferroptosis is selectively inhibited by iron chelators (e.g., deferoxamine) but not by inhibitors of apoptosis or autophagy, supporting its unique signaling mechanism. Thus, iron imbalance is not exclusive to ferroptosis. While ferroptosis remains the most iron-dependent form of cell death and displays the most pronounced amplification of lipid peroxidation, recent studies demonstrate that disturbances in iron homeostasis can also influence pyroptosis and necroptosis (Du et al., 2024). Impaired iron homeostasis is a central upstream determinant of ferroptosis sensitivity. Upregulation of iron transport proteins such as transferrin (Tf), transferrin receptor (TfR), and divalent metal transporter 1 (DMT1) enhances cellular iron uptake, while loss or dysfunction of ferroportin 1 (FPN1) hampers iron efflux, leading to intracellular iron overload and oxidative damage. Experimental data suggest that when serum labile iron exceeds 30 μM, lipid peroxidation levels can increase by over threefold relative to baseline (Tang et al., 2021). Ferritin heavy chain (FTH1) mitigates iron toxicity by sequestering excess free iron. In contrast, nuclear receptor coactivator 4 (NCOA4)-mediated ferritinophagy promotes ferritin degradation and iron release. Loss of NCOA4 substantially decreases ferroptosis susceptibility, reducing cell death by approximately 62% (Savo and Dill, 2019). Moreover, disruption of autophagy-related genes such as Atg5 and Atg7 also suppresses Erastin-induced ferroptosis, indicating the involvement of autophagy-dependent mechanisms in this process (Green, 2019). At the core of ferroptotic execution lies the GSH–GPX4 antioxidant defense system. GPX4 detoxifies lipid hydroperoxides by reducing them to their corresponding alcohols using GSH as a substrate, thereby halting lipid radical chain reactions and preserving membrane integrity. Compounds such as RSL3 irreversibly inhibit GPX4 by binding to its active site, resulting in LPO accumulation and ferroptotic cell death (Fang et al., 2019). The synthesis of GSH depends on System xc^−^, a cystine/glutamate antiporter composed of SLC7A11 and SLC3A2. Inhibitors of this system, such as Erastin, drastically reduce intracellular GSH levels—by over 80%—thereby impairing GPX4 function and triggering ferroptosis (Ayton et al., 2017). Lipid remodeling represents the terminal phase of ferroptosis. Polyunsaturated fatty acids (PUFAs), such as arachidonic acid, are esterified by ACSL4 and incorporated into phospholipid membranes with the aid of LPCAT3, forming peroxidation-prone PUFA-phospholipids. Genetic deletion of ACSL4 or LPCAT3 confers resistance to ferroptosis (Acosta-Cabronero et al., 2016). Additionally, the lipoxygenase enzyme ALOX15 catalyzes the oxidation of PUFA-phosphatidylethanolamines (PUFA-PEs) into toxic lipid peroxides. Inhibition of ALOX15 by compounds like baicalein markedly reduces ferroptosis induced by Erastin (Ayton et al., 2015). Current research has delineated several pivotal axes in ferroptosis regulation: iron homeostasis modulation (e.g., NCOA4/TfR pathway), antioxidant defense (System xc^−^/GPX4 axis), and lipid remodeling (ACSL4/ALOX15 pathway). Targeted pharmacological interventions along these pathways have shown therapeutic promise in models of ischemic brain injury, neurodegeneration, and cancer. These mechanistic insights may pave the way for translational applications of ferroptosis-based therapies in Alzheimer’s disease and related disorders (Figure 1).

### 2.3 Clinical evidence supporting the involvement of iron dysregulation in Alzheimer’s disease progression

“Recent clinical and imaging studies indicate that iron dysregulation is an active contributor to early Alzheimer’s disease (AD) pathogenesis rather than a mere byproduct of late neurodegeneration. Quantitative susceptibility mapping (QSM) shows increased regional brain iron that tracks amyloid-related cognitive decline in longitudinal cohorts (Ayton et al., 2017), with elevated hippocampal iron detectable *in vivo* by MRI (Raven et al., 2013). In biofluid studies, cerebrospinal fluid ferritin predicts AD outcomes and associates with amyloid and tau measures, supporting disrupted iron homeostasis as an independent driver of disease progression (Du et al., 2024). Moreover, higher iron content across cortical/subcortical gray matter and hippocampal subfields correlates with memory decrements even in cognitively normal older adults (Spence et al., 2022). Mechanistically, impaired iron export—e.g., zinc-mediated inhibition of amyloid precursor protein (APP) ferroxidase activity—provides a route to local iron accumulation (Duce et al., 2010), while familial AD presenilin mutations confer selective vulnerability to ferroptosis in human neuronal models (Greenough et al., 2022). Together, these primary data delineate a pathogenic axis spanning focal iron accumulation, peripheral–central iron imbalance, and ferroptosis susceptibility in early AD.” Further evidence shows that ferritin levels in cerebrospinal fluid (CSF) can predict the rate of cognitive decline and are significantly associated with both Aβ-positivity on PET imaging and elevated t-Tau concentrations. These findings support the hypothesis that disrupted iron homeostasis may serve as an independent driver of AD progression (Molinuevo et al., 2018).

In peripheral biomarker studies, AD patients commonly exhibit elevated serum ferritin and reduced levels of soluble transferrin receptor (sTfR), suggesting impaired systemic iron utilization (Chatterjee et al., 2023). Among individuals with mild cognitive impairment (MCI), increased CSF ferritin correlates strongly with reduced Aβ42, elevated t-Tau, and greater degrees of brain atrophy (Spence et al., 2022). A prospective cohort study reported that combining sTfR and ferritin into a ratio-based index achieved an area under the curve (AUC) of 0.87 in predicting Aβ-positivity, outperforming traditional individual biomarkers (Du et al., 2024). Importantly, iron deposition also correlates with quantitative memory decline. In cognitively normal older adults, increased iron content in cortical and subcortical gray matter regions has been significantly associated with reductions in overall memory performance and immediate recall ability (Kahn-Kirby et al., 2019).

### 2.4 Experimental studies elucidate the pathogenic role of ferroptosis in Alzheimer’s disease

A growing body of experimental evidence from animal and cellular models has clarified the mechanistic links between iron dysregulation and ferroptosis in Alzheimer’s disease (AD). In APP/PS1 transgenic mice, hippocampal iron levels begin to rise at 6 months of age, with an increase of up to 48% observed by month 8. This accumulation is accompanied by downregulation of glutathione peroxidase 4 (GPX4), upregulation of acyl-CoA synthetase long-chain family member 4 (ACSL4), and elevated lipid peroxidation—hallmarks of ferroptotic cell death (Taylor et al., 2008). Pharmacological intervention with rosiglitazone, an ACSL4 inhibitor, led to a 44% reduction in 4-hydroxynonenal (4-HNE) levels and a 42% decrease in Aβ plaque deposition in brain tissues. Notably, these biochemical improvements were accompanied by amelioration of cognitive and behavioral deficits, suggesting that ferroptosis is not only pathogenic but also potentially reversible and targetable (Li J. Y. et al., 2024). *In vitro* studies using Aβ42-treated neuronal cultures have demonstrated upregulation of nuclear receptor coactivator 4 (NCOA4), indicating activation of the ferritinophagy pathway. This leads to ferritin degradation, increased labile iron release, elevated lipid peroxidation, and disruption of neuronal membrane integrity. Importantly, either genetic silencing of NCOA4 or treatment with the iron chelator deferoxamine (DFO) significantly attenuated these pathological changes, resulting in over 60% improvement in neuronal survival (Plascencia-Villa and Perry, 2021). Additional studies have revealed that hyperphosphorylated tau protein can directly enhance the transcription of ACSL4, thereby intensifying the activity of the PUFA–phosphatidylethanolamine (PUFA–PE) pathway and accelerating lipid peroxidation. This forms a pathogenic positive feedback loop—linking iron accumulation, Aβ/tau pathology, and ferroptosis—which collectively exacerbate neuronal injury (Yadav et al., 2023; Mohammadi et al., 2024). However, across many checkpoint-focused AD studies that primarily target oxidative stress, Aβ clearance, or neuroinflammatory ‘checkpoints’, the field has only rarely measured a complete ferroptosis signature—that is, simultaneous assessment of iron-dependent lipid peroxidation together with GPX4/SLC7A11 and ACSL4/LPCAT3 alterations, combined with ferroptosis-selective rescue experiments (e.g., ferrostatin-1, liproxstatin-1, or FSP1 activation). To make the current evidence and gaps explicit, we now include Table 2 summarizing representative ferroptosis-in-AD studies (human, animal, and cellular), indicating which hallmarks were examined and whether selective rescue or checkpoint linkage was established (Bao et al., 2021; Greenough et al., 2022; Li et al., 2022; Thorwald et al., 2025). Existing primary data show that although iron overload and oxidative injury are consistently observed, comprehensive ferroptosis validation remains limited, highlighting the need for standardized, hallmark-integrated approaches in future research. Together, these preclinical findings strongly indicate that ferroptosis is not a secondary consequence of neurodegeneration in AD, but rather functions as an upstream pathogenic mechanism that drives Aβ production, tau hyperphosphorylation, and neuronal apoptosis. Ferroptosis likely plays a central role in both the initiation and progression of AD pathology.

**Table 2:** Summary of ferroptosis-in-AD studies and checkpoint linkage.

Study	Model	Markers measured (GPX4/SLC7A11, ACSL4/LPCAT3, LPO, Fe^2+^)	Selective rescue (Fer-1/Lip-1)	Checkpoint linkage	Outcome
Li et al. (2022)	APP/PS1 mouse	GPX4↓ ACSL4↑ LPO↑ Fe^2+^↑	Fer-1 ✓	Iron export defect	Memory ↑
Thorwald et al. (2025)	Human neurons (PSEN mutant)	GPX4↓ ACSL4↑	Fer-1/Lip-1 ✓	Presenilin mutation	Cell death ↓
Boshuizen et al. (2020)	Aβ neurons	GPX4↓ LPO↑ Fe^2+^↑	Fer-1 ✓	Mitochondrial dysfunction	Viability ↑
Rouault (2013)	Human/neuron	GPX4↓ LPO↑ Fe^2+^↑	Fer-1 ✓		

This table helps readers visualize where ferroptosis has been directly demonstrated, which hallmarks remain untested, and how these findings connect (or fail to connect) with checkpoint-related mechanisms in AD.

### 2.5 Clinical prospects of iron metabolism biomarkers in precision diagnosis and therapy

The marked heterogeneity of Alzheimer’s disease (AD) underscores the need for precision medicine approaches grounded in mechanism-specific biomarker systems. As a newly emerging and extensively studied pathway, iron dysregulation is activated early in AD pathogenesis and exhibits substantial cross-talk with classical pathological features such as Aβ deposition and tau hyperphosphorylation—rendering it a promising target for biomarker development. In neuroimaging, quantitative susceptibility mapping (QSM) is increasingly employed as a noninvasive tool for early detection of cognitive impairment in AD. When integrated with PET imaging or electroencephalography, QSM enables simultaneous quantification of brain iron deposition and Aβ burden, facilitating molecular subtype stratification and individualized diagnostic profiling (Boshuizen et al., 2020). Recent advances have integrated QSM analysis with artificial intelligence (AI) technologies—most notably, the QSMNet deep learning algorithm. This integration has significantly improved the spatial resolution and accuracy of automated iron deposition detection, enhancing the clinical applicability of QSM in multicenter AD populations (Rouault, 2013). In the context of liquid biopsy, biomarkers reflecting peripheral iron homeostasis—such as CSF ferritin and the serum sTfR/ferritin ratio—have demonstrated high sensitivity and specificity in distinguishing mild cognitive impairment (MCI) from AD (Du et al., 2024).

Moreover, iron-related biomarkers hold potential for treatment response prediction and therapeutic monitoring. Studies have shown that reductions in CSF ferritin following antioxidant therapy (e.g., N-acetylcysteine) or iron chelation (e.g., deferoxamine) correlate positively with improvements in cognitive performance, supporting their use as dynamic indicators of clinical efficacy (Du et al., 2024). Emerging research efforts aim to integrate brain iron deposition maps with genetic profiles and metabolic phenotypes to construct risk stratification models for AD progression. These integrative frameworks pave the way for personalized prediction algorithms and precision-targeted intervention strategies (Raven et al., 2013). With ongoing advances in multi-omics profiling and machine learning techniques, biomarkers derived from iron metabolism are poised to become central pillars in the development of precision medicine strategies for Alzheimer’s disease.

## 3 Pathogenic mechanisms linking ferroptosis to Alzheimer’s disease

### 3.1 Iron dysregulation bridges immune imbalance and neurodegeneration in Alzheimer’s disease

**Figure 2:**
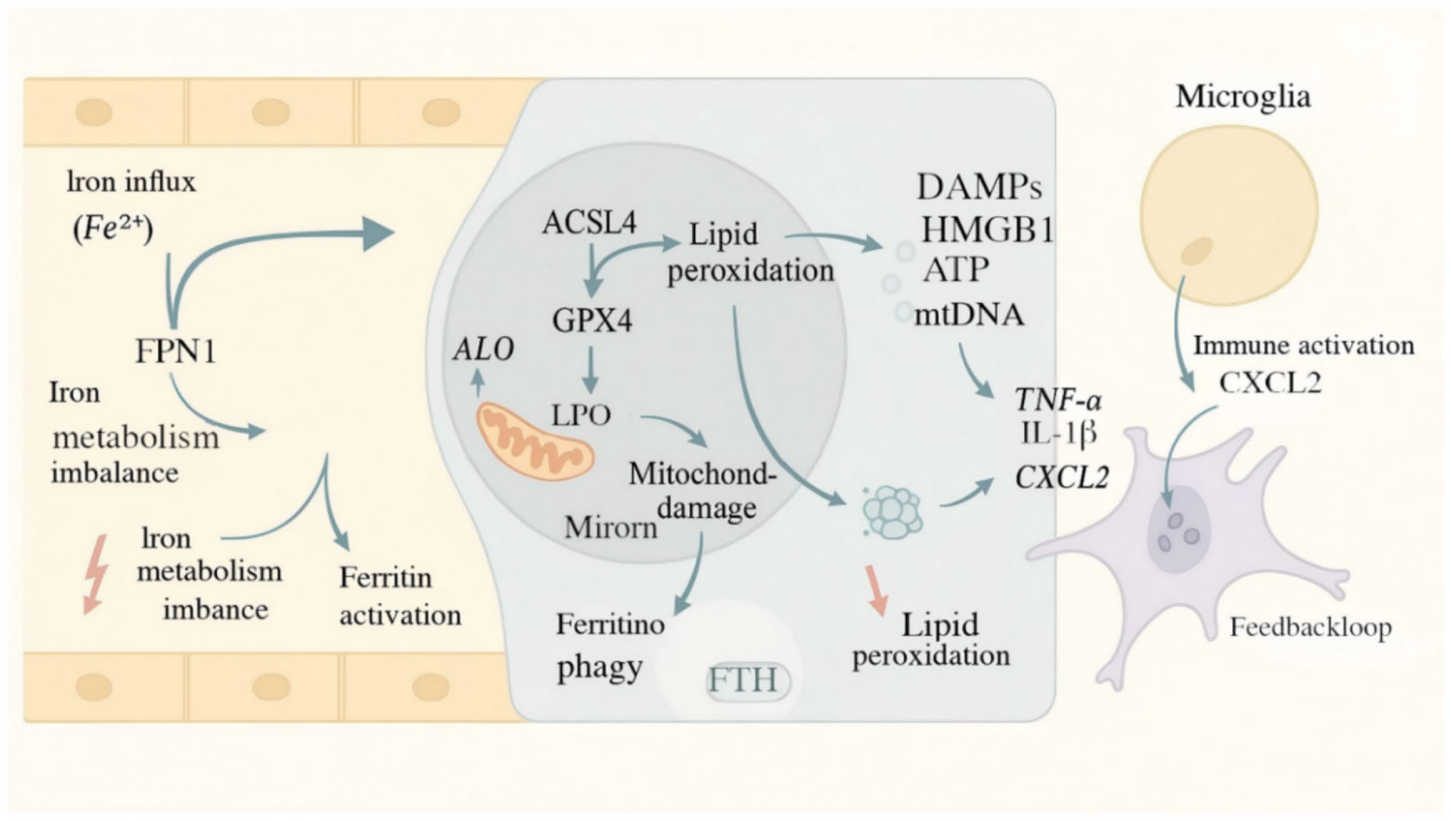
Ferroptosis-driven cascade in Alzheimer’s disease: The iron–lipid–immune axis. This diagram illustrates the molecular mechanism by which ferroptosis contributes to Alzheimer’s disease (AD) through the iron–lipid–immune axis. The schematic is divided into three compartments: the blood–brain barrier (BBB), neuronal cytoplasm, and microglia. Disrupted iron homeostasis leads to excess Fe^2+^ influx through a compromised BBB, initiating ferritinophagy and promoting lipid peroxidation (LPO) accumulation. Glutathione peroxidase 4 (GPX4) inactivation, along with ACSL4 upregulation, facilitates the propagation of lipid oxidation and mitochondrial dysfunction. Damaged neurons release damage-associated molecular patterns (DAMPs) such as HMGB1, ATP, and mtDNA, which activate microglia via TLR4 signaling. Activated microglia secrete proinflammatory cytokines (TNF-α, IL-1β) and chemokine CXCL2, which further induces ACSL4 expression in neurons via CXCR2 signaling, reinforcing the ferroptotic cascade. This feedback loop underscores the cross-talk between ferroptosis and neuroinflammation in AD pathology.

The immune system serves as a pivotal bridge between ferroptosis and neurodegeneration in Alzheimer’s disease (AD). Disruption of iron homeostasis not only triggers intracellular oxidative stress but also activates both central and peripheral immune responses, establishing a “iron–immunity-neurodegeneration” feedback loop that amplifies inflammation and exacerbates neuronal damage. Studies have demonstrated that under iron-overloaded conditions, neurons release damage-associated molecular patterns (DAMPs) such as high mobility group box-1 protein (HMGB1), adenosine triphosphate (ATP), and mitochondrial DNA (mtDNA). These molecules activate immune-sensing pathways in microglia, including TLR4, P2X7, and cGAS–STING, leading to the robust release of pro-inflammatory cytokines such as interleukin-1β (IL-1β) and tumor necrosis factor-α (TNF-α) (Ayton et al., 2013). Activated microglia secrete the chemokine CXCL2, which in turn upregulates ACSL4 expression and suppresses GPX4 activity in adjacent neurons, intensifying lipid peroxidation and membrane damage. This cross-cellular signaling establishes a ferroptosis–microglia–neuron amplification cascade (Duce et al., 2010). In AD animal models, M1-polarized microglia frequently co-exist with iron accumulation, indicating a synergistic interaction between inflammatory activation and disrupted iron metabolism in driving neurotoxicity (Mendoza and Scharff, 2017). Astrocytes also contribute significantly to this pathogenic process. These glial cells secrete Galectin-9, which interacts with CD47 on microglia to suppress their phagocytic capacity, thereby facilitating Aβ immune evasion and sustaining neuroinflammation (Wang et al., 2010). Under iron overload, astrocytes exhibit markedly increased CD47 expression, reinforcing a pathological loop of immune escape, persistent activation, and inflammatory spread (Pape et al., 2021). Simultaneously CD8^+^ T cell infiltration is significantly elevated in AD brain tissues. Upon activation, these T cells release interferon-γ (IFN-γ), which downregulates neuronal expression of ferritin heavy chain (FTH1), resulting in increased intracellular labile iron, thereby enhancing susceptibility to ferroptosis. This may further promote Aβ aggregation and tau pathology (Kamath et al., 2022). Collectively, these findings suggest that iron dyshomeostasis orchestrates immune dysfunction through multiple converging pathways—including microglial and astrocytic activation and central T cell infiltration. This ultimately amplifies neuroinflammation and forms a closed-loop network of “ferroptosis–immune response–neuronal injury,” offering a novel pathophysiological perspective for AD (Figure 2).

### 3.2 Exosome-mediated intercellular propagation of Ferroptotic signaling in Alzheimer’s disease

**Figure 3:**
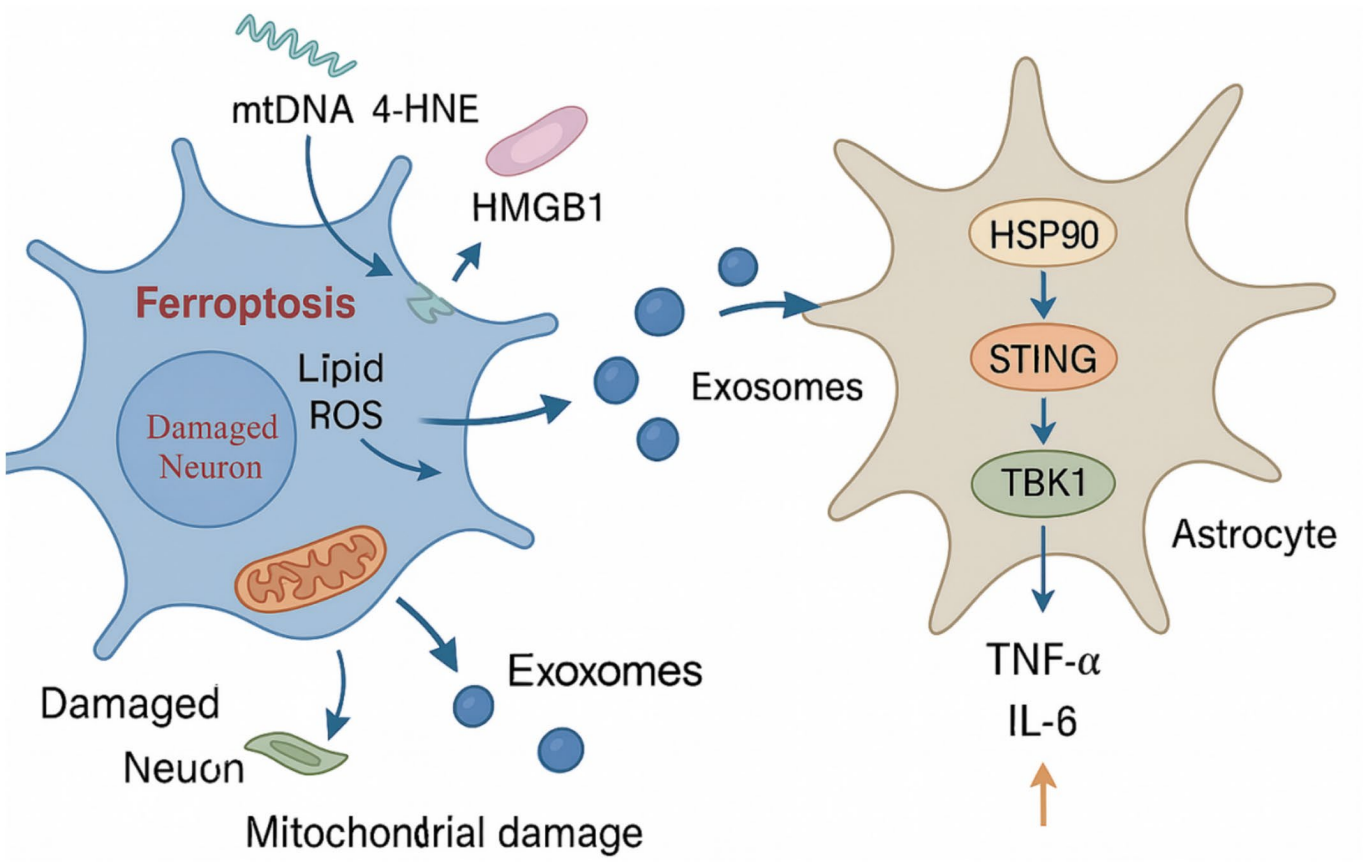
Intercellular propagation of ferroptosis via exosome-mediated signaling. This diagram illustrates the mechanism by which ferroptotic neurons communicate with astrocytes through exosome-mediated signaling. Neuronal ferroptosis is characterized by lipid peroxidation and GPX4 inactivation, resulting in the release of mitochondrial DNA (mtDNA), 4-hydroxynonenal (4-HNE), and HMGB1. These stress-associated molecules are packaged into exosomes and secreted into the extracellular space. Upon uptake by astrocytes, the exosomal cargo activates the HSP90–STING–TBK1 signaling pathway, leading to the upregulation of proinflammatory cytokines such as TNF-α and IL-6. This intercellular crosstalk highlights the role of ferroptosis in propagating neuroinflammatory responses through exosome-based communication.

Exosomes, as pivotal intercellular communication vehicles in the nervous system, play an essential role in transmitting ferroptosis-associated signals and facilitating the spread of pathological stress. Under ferroptotic stress, neurons actively release exosomes enriched with bioactive molecules, including lipid peroxidation–associated enzymes such as ACSL4 and ALOX15, as well as multiple inflammation-regulating microRNAs, such as miR-124-3p (Jiang et al., 2023; Brozek-Mucha and Zdeb, 2018). These vesicles act as carriers to disseminate ferroptotic signals to neighboring cells. Studies have shown that ACSL4-enriched exosomes can be phagocytosed by microglia, leading to the activation of the nuclear factor kappa-B (NF-κB) signaling pathway and polarization of microglia toward a proinflammatory M1 phenotype, thereby amplifying neuroinflammation (He et al., 2024). In parallel, miR-124-3p–containing exosomes can engage the STING–TBK1–IRF3 signaling axis, inducing the production of type I interferons and further enhancing immune activation in the central nervous system (Hu et al., 2022). Beyond lipid enzymes and microRNAs, exosomes derived from ferroptotic neurons also carry immunogenic DAMPs, which can be internalized by both microglia and astrocytes, triggering their innate immune recognition systems and initiating secondary immune responses. This forms a positive feedback loop involving ferroptosis–exosome release–glial activation–neuroinflammation (Li et al., 2025; Murao et al., 2021). This exosome-based signaling cascade transforms ferroptosis from a localized neuronal death event into a spatially expanding pathological phenomenon within neural networks. Through the “exosome–glial–inflammation cascade,” this mechanism facilitates the spread of focal neuronal injury to adjacent brain regions, thereby accelerating region-specific neurodegeneration and functional impairment in Alzheimer’s disease (Figure 3).

### 3.3 Single-cell evidence and limitations of checkpoint–ferroptosis interactions in human AD

Notably, no direct single-cell evidence yet confirms checkpoint regulation of ferroptosis within human Alzheimer’s disease (AD) brains. Current single-nucleus RNA-seq datasets from human AD tissue reveal distinct ferroptosis-related transcriptional programs in glial cells, including upregulation of TFRC, FTH1, SLC40A1, and ACSL4, which indicate enhanced iron turnover and lipid peroxidation susceptibility (Leng et al., 2021). However, PD-1 and PD-L1 transcripts are expressed only at very low levels in these datasets, limiting direct inference of checkpoint-mediated ferroptosis regulation in AD. Mechanistic insights from non-AD neuroinflammatory systems provide complementary evidence: interferon-γ (IFN-γ) robustly induces PD-L1 expression in human astrocytes and microglia, establishing a checkpoint–redox interface that suppresses excessive oxidative injury (Smith et al., 2023; Linnerbauer et al., 2023; Gao et al., 2022). Together, these findings suggest that checkpoint activation could indirectly influence ferroptosis-associated pathways in AD, yet the connection remains inferential, underscoring the need for future multi-omics and spatial single-cell studies in human brains to validate this link.

### 3.4 Checkpoint dysregulation couples immune escape and ferroptosis in Alzheimer’s disease

Immune checkpoints play critical regulatory roles in maintaining immune homeostasis in the central nervous system (CNS) and facilitating the clearance of pathological proteins, such as amyloid-β (Aβ) and hyperphosphorylated tau. Recent studies have uncovered that certain immune checkpoint molecules also directly modulate ferroptosis-related pathways, forming a crucial interface linking iron homeostasis, immune responses, and neuronal injury (Greenough et al., 2022).

In AD models, the CD47–SIRPα axis is markedly activated. CD47 is primarily expressed on neurons and astrocytes, whereas its receptor SIRPα is located on microglia. By emitting a “do not eat me” signal, CD47 inhibits the phagocytic capacity of microglia toward both ferroptotic neurons and Aβ plaques, leading to an immune-tolerant state. This suppression contributes to the accumulation of toxic protein aggregates and aggravates immune imbalance (Lehrman et al., 2018; Hu et al., 2025). In addition to CD47, programmed death ligand-1 (PD-L1) expression is upregulated in AD brain tissues and positively correlates with tissue iron overload (Wu et al., 2022). PD-L1 can activate the STAT3/Nrf2 pathway, leading to enhanced GPX4 expression and exerting a negative feedback effect on ferroptosis (Dai et al., 2025). However, under chronic inflammatory conditions, PD-L1 may also suppress T cell function and promote immune evasion, thereby impairing the clearance of pathological proteins such as Aβ and further exacerbating the neurodegenerative burden (Rosenzweig et al., 2019). Collectively, immune checkpoints exhibit dual roles in the progression of AD. On one hand, they regulate ferroptosis and mitigate oxidative stress and cellular injury; on the other hand, they may contribute to immune escape, promoting pathological protein accumulation and neuronal dysfunction. The imbalance of immune checkpoint signaling likely serves as a critical nexus in the cross-talk between ferroptosis and immune dysregulation, highlighting these molecules as potential therapeutic targets for combinatorial interventions targeting the ferroptosis–immunity axis in AD.

### 3.5 Ferroptosis-induced disruption of the blood–brain barrier in Alzheimer’s disease

**Figure 4:**
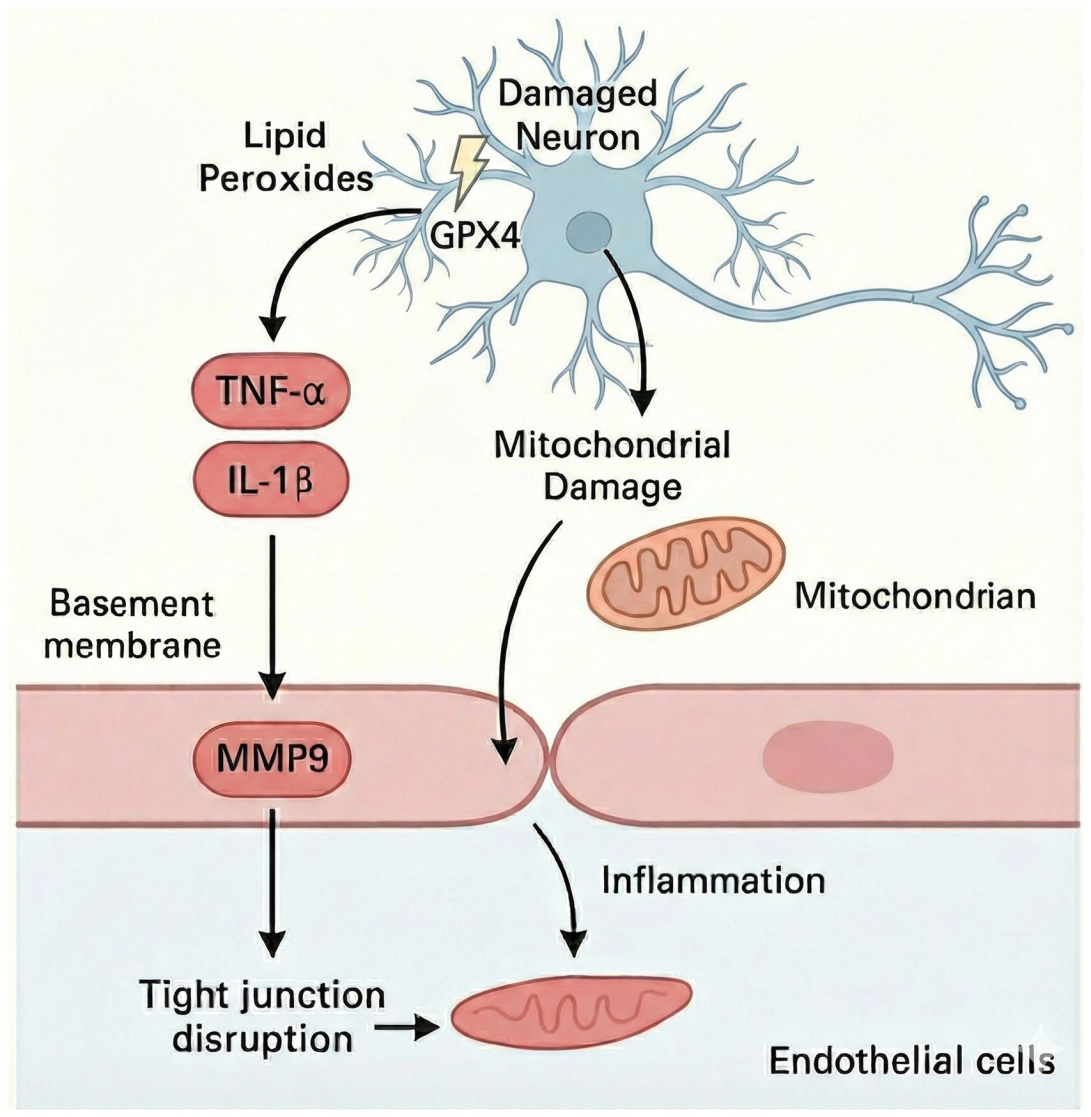
Ferroptosis-induced disruption of the blood–brain barrier. This schematic illustrates how neuronal ferroptosis compromises the integrity of the blood–brain barrier (BBB). Ferroptosis in neurons, marked by GPX4 inactivation and lipid peroxide accumulation, leads to mitochondrial dysfunction and the release of proinflammatory cytokines such as TNF-α and IL-1β. These inflammatory mediators upregulate matrix metalloproteinase 9 (MMP9) expression in adjacent endothelial cells. MMP9 disrupts tight junction proteins and degrades the basement membrane, contributing to increased BBB permeability and neuroinflammation. This mechanism highlights ferroptosis as a key driver of vascular dysfunction in neurodegenerative conditions.

The blood–brain barrier (BBB) is a critical structural interface that maintains central nervous system (CNS) homeostasis by restricting the translocation of blood-derived substances, inflammatory mediators, iron ions, and immune cells into the brain parenchyma through its tight junction architecture. Recent evidence indicates that iron dyshomeostasis and ferroptotic processes directly compromise BBB integrity, thereby facilitating the influx of pathological factors and promoting neuroinflammatory propagation, which accelerates the progression of Alzheimer’s disease (AD) (Xu et al., 2024; Jamrus et al., 2025). On one hand, iron overload markedly enhances the accumulation of reactive oxygen species (ROS) in cerebral endothelial cells, leading to downregulation of ferroportin 1 (FPN1) and impaired iron efflux. This results in local iron deposition and persistent oxidative stress within the cerebrovascular microenvironment (Sato et al., 2022; Long et al., 2023). On the other hand, iron-triggered lipid peroxidation activates matrix metalloproteinase-9 (MMP-9), which degrades key tight junction proteins such as ZO-1 and occludin, thereby disrupting endothelial barrier structure and increasing vascular permeability (Kim et al., 2022). Moreover, under ferroptotic stress, both microglia and astrocytes release proinflammatory cytokines including TNF-α and IL-6, triggering localized vascular inflammation and further compromising BBB function (Kim et al., 2022). Additional studies have reported that elevated brain iron levels lead to increased expression of programmed death ligand-1 (PD-L1) within the neurovascular unit. This PD-L1 upregulation suppresses perivascular T cell activity, impairing their ability to clear pathological signals and enabling ferroptotic mediators to accumulate and disseminate across the BBB (Kim et al., 2024). Taken together, ferroptosis is not only a form of regulated necrotic cell death, but also a potent disruptor of the BBB’s anatomical and immunological defenses. By undermining barrier integrity, ferroptosis establishes a multilevel cascade involving iron overload, BBB permeability, and immune dysregulation, ultimately contributing to AD pathogenesis (Figure 4).

## 4 Therapeutic strategies targeting ferroptosis in Alzheimer’s disease

### 4.1 Therapeutic strategies targeting iron homeostasis

**Figure 5:**
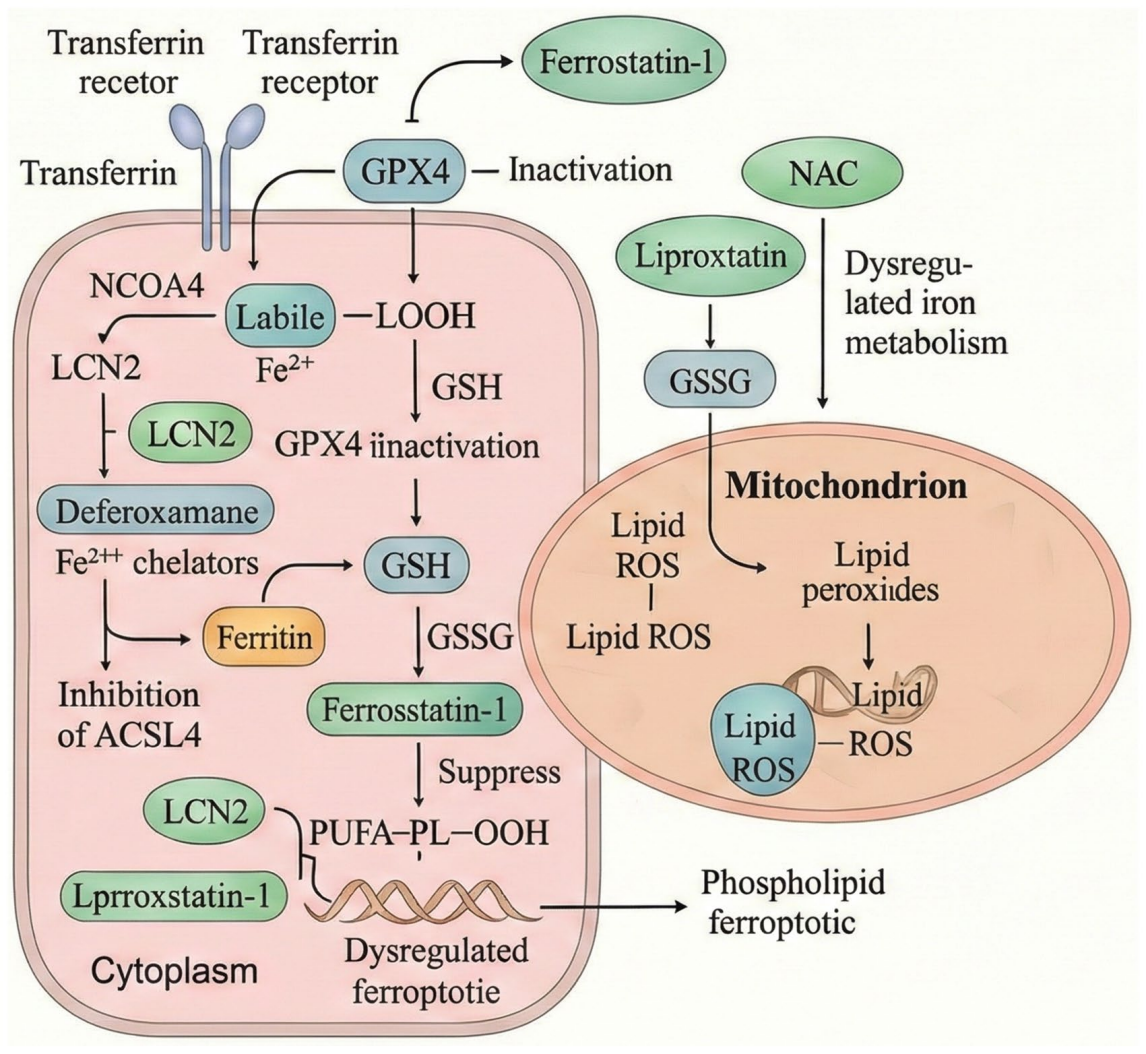
Multi-targeted therapeutic interventions against ferroptosis. This schematic illustrates a comprehensive intervention network targeting key molecular events in ferroptosis, including iron overload, GPX4 inactivation, and lipid peroxidation. Within the cytoplasm, labile Fe^2+^ accumulation is regulated by transferrin–transferrin receptor uptake and LCN2 modulation, while iron chelators (e.g., Deferoxamine) reduce iron-induced oxidative damage. NCOA4-mediated ferritinophagy contributes to ferritin degradation and iron release. GPX4 inactivation, exacerbated by glutathione (GSH) depletion, drives lipid hydroperoxide (LOOH) buildup. In the mitochondria, lipid ROS amplify oxidative stress and promote phospholipid peroxidation. Agents such as Ferrostatin-1, Liproxstatin-1, NAC, and Vitamin E counteract these processes by preserving GSH levels, inhibiting lipid peroxidation, and stabilizing redox homeostasis. Targeting PUFA-PL-OOH and ACSL4 further blocks ferroptotic damage, making these nodes attractive for neuroprotective therapy.

In AD, the disruption of iron homeostasis is considered a pivotal upstream event that triggers ferroptosis activation and drives pathological spread (Figure 5). As such, restoring cerebral iron balance represents a core therapeutic approach for ferroptosis modulation. Currently, iron chelators remain the most direct pharmacological agents for controlling brain iron overload. Representative compounds include deferoxamine (DFO), deferiprone (DFP), and the novel metal-modulating molecule PBT2 (He et al., 2024; Xiao et al., 2024). Among these, DFO has shown efficacy in reducing free iron levels in brain tissue; however, its strong hydrophilicity and poor lipid solubility result in limited blood–brain barrier (BBB) permeability and suboptimal clinical performance. In contrast, DFP possesses favorable lipid solubility and excellent BBB penetration, demonstrating more pronounced neuroprotective effects in AD animal models (Xi et al., 2022; Daglas et al., 2023). PBT2 acts by modulating metal–protein interaction networks, thereby restoring iron–copper balance in the brain and indirectly reducing Aβ deposition, offering dual-target therapeutic potential (Chen L. et al., 2022). “Although numerous preclinical studies have shown that iron chelators such as deferoxamine (DFO), deferiprone (DFP), and PBT2 effectively mitigate brain iron overload, reduce amyloid burden, and improve cognition in AD models, translation to the clinic has proven challenging. Despite their strong iron-binding capacity, classical chelators have limited success in human studies. DFO, while effective in animal models, exhibits poor blood–brain barrier (BBB) permeability, necessitating intranasal or intracerebroventricular delivery to achieve therapeutic concentrations (Guo et al., 2013). DFP and deferasirox (DFX) display improved BBB penetration but carry potential hepatotoxicity and agranulocytosis risks, restricting long-term use (Devos et al., 2014). More recently, two randomized controlled trials of DFP in amyloid-confirmed early AD produced paradoxical outcomes: patients receiving DFP deteriorated faster than those on placebo over 12 months (Lannfelt et al., 2008). Similarly, the phase IIa PBT2 trial demonstrated acceptable safety and modest biomarker modulation without significant clinical efficacy (Ayton et al., 2025). Collectively, these findings indicate that non-selective or excessive chelation of brain iron may disrupt physiological metallostasis, emphasizing the need for precisely titrated, brain-targeted anti-ferroptotic interventions with balanced efficacy–toxicity profiles.” Beyond traditional chelators, several natural compounds have also shown potential in regulating iron transport proteins. For instance, extracts from Guizhi Fuling Pills and Paeonia bark gum (Baikejiao) have been reported to downregulate transferrin receptor 1 (TfR1) and upregulate ferroportin 1 (FPN1) expression, thereby facilitating the restoration of intracellular and extracellular iron equilibrium (Liu R. Z. et al., 2022; Zhang et al., 2019). Notably, microRNAs (miRNAs) have emerged as novel molecular tools for iron regulation. Studies have shown that miR-124 and miR-212 can, respectively, target key regulatory proteins such as FPN1 and iron regulatory protein 2 (IRP2), modulating both iron export and storage capacity in neural cells. These findings provide a mechanistic foundation for developing RNA-based therapeutic interventions (Bao et al., 2020; Zheng and Zhang, 2023). In summary, pharmacological strategies centered on iron homeostasis are evolving from single-agent chelators to multi-target approaches involving small molecules, natural product extracts, and RNA technologies. These developments offer new opportunities for precision modulation and combinatorial therapies in ferroptosis-related neurodegenerative conditions. Future development of iron-targeting therapeutics should prioritize agents with verified blood–brain barrier permeability, minimal systemic toxicity, and the capacity to restore both iron homeostasis and redox balance. In parallel, clinical translation should incorporate mechanistic biomarkers and patient stratification to balance efficacy and safety, enabling the identification of subgroups most likely to benefit from ferroptosis-targeted interventions.

### 4.2 Therapeutic strategies targeting lipid peroxidation and GPX4 protection

Lipid peroxidation represents a central pathogenic event in ferroptosis, contributing to plasma membrane rupture and mitochondrial dysfunction. Its progression is closely linked to the activity and stability of GPX4, making the inhibition of lipid peroxidation and the preservation of GPX4 function pivotal targets in ferroptosis-based therapeutic interventions. On one hand, various antioxidants have been shown to either scavenge lipid peroxyl radicals (LOO•) or maintain intracellular glutathione (GSH) levels, thereby indirectly enhancing GPX4 stability and enzymatic activity. Among them, vitamin E analogs such as α-tocopherol act as classical LOO• scavengers, effectively interrupting radical chain reactions within polyunsaturated fatty acid (PUFA)-rich membrane domains. Coenzyme Q10 (CoQ10), in addition to its antioxidative role, contributes to mitochondrial membrane potential stabilization, improving GPX4 efficiency within mitochondrial membranes (Zhao et al., 2021; Wang F. et al., 2022; McLachlan et al., 1993; Guo et al., 2013). On the other hand, with the deepening of ferroptosis research, specific GPX4 inhibitors such as RSL3 have been widely utilized as inducers in mechanistic models. Meanwhile, several newly developed GPX4 stabilizers and expression enhancers—such as KCF18 and ebselen—have shown neuroprotective effects in experimental settings (SharathBabu et al., 2025; Conrad and Proneth, 2020).

Furthermore, nuclear factor erythroid-2-related factor 2 (Nrf2), a key transcriptional regulator of both GSH biosynthesis and GPX4 expression, is garnering increasing attention. Its pharmacological activators, including oltipraz and bardoxolone methyl, have been shown to ameliorate cognitive deficits and attenuate lipid peroxidation damage in AD models (Chu et al., 2024). Collectively, therapeutic frameworks targeting the lipid peroxidation cascade—ranging from free radical scavenging and GPX4 enhancement to the modulation of the Nrf2–GSH signaling axis—are gradually being established. These strategies are anticipated to work synergistically with iron homeostasis regulators, offering a combinatorial approach to mitigating ferroptosis-associated neurodegeneration.

### 4.3 Immunomodulatory strategies for ferroptosis regulation in Alzheimer’s disease

In Alzheimer’s disease (AD), ferroptosis is not only characterized by disrupted iron metabolism and lipid peroxidation, but also by the activation of immune cells including microglia, astrocytes, and T lymphocytes. These processes converge into a pathological network defined by the synergistic amplification of iron dysregulation, inflammation, and immune dysfunction. Thus, targeting immune pathways represents a promising strategy to suppress ferroptosis-mediated neurodegeneration. Studies have shown that TREM2 (triggering receptor expressed on myeloid cells 2), a surface receptor on microglia, plays a pivotal role in regulating their polarization status. TREM2 agonists, such as AL002, promote microglial transformation toward the anti-inflammatory M2 phenotype, thereby reducing the release of pro-inflammatory M1 cytokines (e.g., TNF-α, IL-1β) and indirectly mitigating inflammation-driven ferroptotic cascades. These interventions have shown potential in improving AD-related cognitive impairment (Huang et al., 2023; Melchiorri et al., 2023). Cytokines such as IL-1β and TNF-α also upregulate ACSL4 expression in neurons, promoting PUFA oxidation and enhancing lipid peroxidation, which increases neuronal susceptibility to ferroptosis. Neutralizing antibodies against these cytokines have been shown to delay ferroptosis amplification and provide neuroprotection in animal models, indicating potential clinical applicability (Deng et al., 2023). At the astrocyte level, the Galectin-9/CD47 axis has been implicated in immune evasion. Increased Galectin-9 expression activates CD47 signaling, which inhibits immune cell phagocytosis of damaged or ferroptotic cells. This facilitates the escape and dissemination of ferroptotic signals from immune clearance. Blocking this axis may restore immunosurveillance, thereby halting pathological signal propagation (Steelman and Li, 2014; Lee et al., 2024). More notably, substantial CD8^+^ T-cell infiltration and aberrant PD-L1 signaling have been observed in AD brain tissues, suggesting the presence of a locally immunosuppressive microenvironment. Although immune checkpoint inhibitors like PD-L1 antibodies have proven effective in cancer immunotherapy, their applicability in AD remains under investigation. Whether releasing immunosuppression could facilitate the clearance of ferroptosis-associated pathogenic signals is an emerging research direction (Hu and Weiner, 2024; Wu et al., 2022). In summary, from modulating microglial polarization and neutralizing pro-inflammatory cytokines, to targeting immune checkpoints and astrocyte-derived inhibitory pathways, immunomodulatory strategies offer multifaceted approaches to disrupt the ferroptosis–inflammation interplay. These interventions open new avenues for therapeutic development in AD management.

### 4.4 Therapeutic strategies targeting the ferroptosis–blood–brain barrier disruption axis in Alzheimer’s disease

A growing body of evidence indicates a pathological interplay between ferroptosis and blood–brain barrier (BBB) dysfunction in Alzheimer’s disease (AD). The iron-overload state commonly observed in AD brains exacerbates neuronal oxidative damage and, through the release of pro-inflammatory mediators and activation of immune cells, contributes to the structural disruption of the BBB. This forms a vicious cycle of “iron overload → inflammation → BBB damage → increased iron permeability,” further aggravating disease progression. Studies have demonstrated that ferroptosis-induced immune activation downregulates the expression of key tight junction proteins (e.g., ZO-1, Occludin) in brain endothelial cells. This impairs capillary integrity, allowing peripheral iron, lymphocytes, and cytokines to infiltrate the brain parenchyma, thereby amplifying local inflammation and iron dysregulation (Chen X. et al., 2022; Jamrus et al., 2025). One effective intervention involves the use of iron chelators, which not only eliminate excess labile iron but also partially restore BBB structural proteins. For example, deferiprone (DFP) and PBT2 have been shown to increase endothelial tight junction protein expression in AD models, reduce vascular leakage, and improve BBB integrity, suggesting an indirect BBB-protective potential (Ayton et al., 2025).

Moreover, ferroptosis-associated damage molecules such as high mobility group box-1 (HMGB1) and chemokine CXCL2 can directly impair the functional stability of brain microvascular endothelial cells. Targeting these molecules with neutralizing antibodies or small-molecule antagonists (e.g., glycyrrhizin) has demonstrated BBB-stabilizing effects *in vivo*, including reductions in brain edema and vascular permeability (Lochhead et al., 2020; Festoff et al., 2016; Nishibori et al., 2020; Yang et al., 2024). Importantly, the BBB functions as the core of the neurovascular unit (NVU), whose integrity depends on the coordinated homeostasis of surrounding neurons, astrocytes, and the basal lamina. A recent study showed that PUFA-enriched neurons under ferroptotic stress are more likely to trigger lipid peroxidation (LPO) and structural disruption in adjacent endothelial cells. This suggests that enhancing the antioxidant and anti-ferroptotic capacity of perivascular tissues may also be a viable strategy to preserve BBB function (Zhao Y. et al., 2023).

In summary, interventions targeting the ferroptosis–BBB axis—including iron chelation, blockade of inflammatory mediators, and reinforcement of NVU antioxidant capacity—offer dual-level strategies at both structural and molecular dimensions for therapeutic advancement in AD.

### 4.5 Targeting mitochondrial function and intercellular ferroptosis propagation in Alzheimer’s disease

Mitochondria serve as a central hub for cellular energy metabolism and lipid peroxidation, playing a pivotal role in the amplification and integration of ferroptosis-related signals. In the brains of Alzheimer’s disease (AD) patients, pathological features such as reduced mitochondrial membrane potential (ΔΨm), accumulation of mitochondrial peroxides, aberrant opening of the mitochondrial permeability transition pore (mPTP), and mitochondrial iron (mFe) overload are commonly observed—each closely linked to ferroptosis induction (Chen et al., 2023; Costa et al., 2023). Thus, therapeutic strategies targeting mitochondrial structural integrity and metabolic regulation have emerged as key approaches to attenuate ferroptotic progression. Mitochondria-targeted antioxidants, such as MitoQ and SkQ1, have been shown to scavenge mitochondrial reactive oxygen species (ROS), preserve membrane integrity, and maintain redox homeostasis, ultimately mitigating neuronal death and cognitive decline in multiple AD models (Young and Franklin, 2019; Stefanova et al., 2016). From the perspective of mitochondrial iron metabolism, iron-export transporters such as ferroportin 1 (FPN1) and ATP-binding cassette subfamily B member 8 (ABCB8) are crucial for maintaining mFe homeostasis. Overexpression of FPN1 in neurons significantly reduces mitochondrial iron accumulation, alleviates lipid peroxidation, and improves pathological phenotypes in AD models (Bao et al., 2024; Guo et al., 2024). Importantly, ferroptosis is not confined to individual cells but can propagate intercellularly within the neural microenvironment. Neurons undergoing ferroptosis release damage-associated molecular patterns (DAMPs) such as high-mobility group box-1 protein (HMGB1), ATP, and exosomes enriched in ACSL4-derived metabolites. These DAMPs can be sensed by microglia, triggering the cGAS–STING pathway and promoting the release of pro-inflammatory cytokines including TNF-α and IL-1β, which may drive adjacent cells into a ferroptotic state (Gao et al., 2023; Ru et al., 2024). Moreover, astrocytes in AD often exhibit impaired phagocytic capacity and defective immune clearance, resulting in the accumulation of neuronal debris and secondary iron release, further exacerbating local cytotoxicity and iron dyshomeostasis (Zeng et al., 2025). Based on these mechanisms, emerging interventional strategies have been proposed to curb intercellular ferroptotic spread, including: blocking exosome-mediated signal transmission, inhibiting STING pathway activation, and enhancing astrocytic phagocytic and clearance functions. Collectively, these approaches offer novel mechanistic insights and therapeutic avenues for targeting ferroptosis propagation in the AD brain.

## 5 Challenges and future perspectives

In recent years, ferroptosis has gained increasing recognition as a form of regulated cell death implicated in the pathogenesis of Alzheimer’s disease (AD). However, from basic mechanistic studies to translational applications, numerous critical challenges and unresolved questions remain. First, the spatiotemporal dynamics of ferroptosis activation during AD progression remain poorly characterized. The patterns and timing of ferroptotic activation across distinct brain regions—such as the hippocampus, cortex, and globus pallidus—as well as in different cell types including neurons, microglia, and astrocytes, lack systematic annotation and multimodal profiling. High-resolution “ferroptosis atlases” integrating emerging technologies such as single-cell omics, spatial transcriptomics, and MRI–mass spectrometry fusion platforms are urgently needed to delineate these dynamics (Currais et al., 2025). Second, the causal relationships between ferroptosis and classical AD pathologies—including Aβ deposition, tau hyperphosphorylation, and chronic neuroinflammation—remain unclear. Most current studies are limited to correlative observations, lacking definitive upstream–downstream regulatory frameworks, thereby hindering target identification and precise therapeutic window determination (Zhao D. et al., 2023). Notably, the majority of checkpoint-oriented AD models still lack measurement of ferroptosis-defining hallmarks or validation using ferroptosis-selective rescue paradigms (see Table 2). Third, existing ferroptosis-targeted strategies rely heavily on preclinical animal models, and clinically translatable agents with brain specificity and low toxicity are still scarce. For instance, although GPX4 activators and ACSL4 inhibitors show efficacy *in vitro*, their pharmacokinetic and pharmacodynamic properties within the human brain microenvironment remain to be systematically evaluated (Majernikova et al., 2021). In addition, ferroptosis acts as a systemic stress response, capable of propagating intercellularly through mechanisms such as exosome-mediated signaling, phagocytic dysfunction, and immune evasion. Yet, the cell-to-cell propagation model of ferroptosis in complex brain disorders like AD remains largely unexplored, representing a crucial frontier for future investigation. Looking ahead, several key directions may accelerate the transition of ferroptosis research from basic science to clinical translation in AD:

Construction of a dynamic, spatiotemporal ferroptosis atlas through integrated multi-omics profiling to define pathological staging and regional specificity; Establishment of comprehensive mechanistic models that incorporate mitochondrial oxidative stress, lipid metabolic reprogramming, and inflammatory amplification, to enable pathway-synergistic targeting; Development of novel drug delivery systems with effective blood–brain barrier (BBB) penetration to improve the specificity and stability of molecular interventions; Application of artificial intelligence, bioinformatics, and systems biology to construct regulatory ferroptosis networks and uncover combinatorial, multi-targeted therapeutic nodes. In conclusion, ferroptosis research in AD is entering a critical translational phase. Driven by technological innovation and mechanistic integration, this field holds great promise as a precision intervention strategy for the prevention and treatment of Alzheimer’s disease.

## 6 Conclusion

Ferroptosis, a regulated form of cell death driven by iron accumulation and lipid peroxidation, has been increasingly recognized as a critical contributor to the pathogenesis and progression of Alzheimer’s disease (AD). This process involves a complex pathological network encompassing disrupted iron homeostasis, GPX4 inactivation, abnormal lipid metabolism, mitochondrial dysfunction, and amplified immune-inflammatory responses. In the early stages of AD, ferroptosis may act as a co-trigger of Aβ aggregation and tau hyperphosphorylation by promoting neuronal degeneration, driving microglial polarization, and compromising the integrity of the blood–brain barrier (BBB). As the disease advances, ferroptosis-induced release of damage-associated molecular patterns (DAMPs), exosomal transmission, and immune evasion mechanisms further amplify neuroinflammation and cell damage, establishing a cascade of intercellular pathological propagation that accelerates neurodegeneration. To date, several key ferroptosis-related targets—such as GPX4, ACSL4, FTH1, and SLC7A11—and upstream regulatory pathways including Nrf2, TLR4, and STING, have been identified. A number of small-molecule inhibitors and bioactive compounds from traditional Chinese medicine have shown promising neuroprotective effects in AD animal models, laying a theoretical and experimental foundation for ferroptosis-targeted therapeutic strategies. Looking forward, future research should prioritize: (1) constructing a spatiotemporal map of ferroptosis across AD pathological stages; (2) clarifying causal relationships between ferroptosis and classical AD hallmarks; and (3) developing brain-targeted, biocompatible ferroptosis modulators. Integration of artificial intelligence, bioinformatics, and systems biology approaches will further aid in identifying multi-target intervention nodes and optimizing treatment strategies. With advances in mechanistic understanding and translational tools, ferroptosis is poised to become a promising therapeutic breakthrough in the precision treatment of Alzheimer’s disease.
